# Mechanistic insights into the conversion of flavin adenine dinucleotide (FAD) to 8-formyl FAD in formate oxidase: a combined experimental and in-silico study

**DOI:** 10.1186/s40643-024-00782-4

**Published:** 2024-07-10

**Authors:** Kai Wen, Sirui Wang, Yixin Sun, Mengsong Wang, Yingjiu Zhang, Jingxuan Zhu, Quanshun Li

**Affiliations:** 1 Key Laboratory for Molecular Enzymology and Engineering of Ministry of Education, School of Life Sciences, Jilin University, Changchun, 130012 China; 2 Center for Supramolecular Chemical Biology, Jilin University, Changchun, 130012 China

**Keywords:** Formate oxidase, 8-Formyl flavin adenine dinucleotide, Oxidative maturation, Molecular dynamics simulation, QM/MM umbrella sampling simulation

## Abstract

Formate oxidase (FOx), which contains 8-formyl flavin adenine dinucleotide (FAD), exhibits a distinct advantage in utilizing ambient oxygen molecules for the oxidation of formic acid compared to other glucose-methanol-choline (GMC) oxidoreductase enzymes that contain only the standard FAD cofactor. The FOx-mediated conversion of FAD to 8-formyl FAD results in an approximate 10-fold increase in formate oxidase activity. However, the mechanistic details underlying the autocatalytic formation of 8-formyl FAD are still not well understood, which impedes further utilization of FOx. In this study, we employ molecular dynamics simulation, QM/MM umbrella sampling simulation, enzyme activity assay, site-directed mutagenesis, and spectroscopic analysis to elucidate the oxidation mechanism of FAD to 8-formyl FAD. Our results reveal that a catalytic water molecule, rather than any catalytic amino acids, serves as a general base to deprotonate the C8 methyl group on FAD, thus facilitating the formation of a quinone-methide tautomer intermediate. An oxygen molecule subsequently oxidizes this intermediate, resulting in a C8 methyl hydroperoxide anion that is protonated and dissociated to form OHC-RP and OH^−^. During the oxidation of FAD to 8-formyl FAD, the energy barrier for the rate-limiting step is calculated to be 22.8 kcal/mol, which corresponds to the required 14-hour transformation time observed experimentally. Further, the elucidated oxidation mechanism reveals that the autocatalytic formation of 8-formyl FAD depends on the proximal arginine and serine residues, R87 and S94, respectively. Enzymatic activity assay validates that the mutation of R87 to lysine reduces the *k*_cat_ value to 75% of the wild-type, while the mutation to histidine results in a complete loss of activity. Similarly, the mutant S94I also leads to the deactivation of enzyme. This dependency arises because the nucleophilic OH^−^ group and the quinone-methide tautomer intermediate are stabilized through the noncovalent interaction provided by R87 and S94. These findings not only explain the mechanistic details of each reaction step but also clarify the functional role of R87 and S94 during the oxidative maturation of 8-formyl FAD, thereby providing crucial theoretical support for the development of novel flavoenzymes with enhanced redox properties.

**Supplementary Information:**

The online version contains supplementary material available at 10.1186/s40643-024-00782-4.

## Introduction

Formic acid, characterized by its high gravimetric energy density and substantial reduction equivalents, is a fundamental raw material in the chemical industry. Formate oxidase (FOx) from *Aspergillus oryzae* is a formate-specific flavoprotein oxidase that belongs to the glucose-methanol-choline (GMC) oxidoreductase superfamily (Doubayashi et al. 2011; Maeda et al. 2009a). FOx exhibits considerable biocatalytic potential by efficiently utilizing ambient oxygen to oxidize formic acid, concurrently producing hydrogen peroxide (H_2_O_2_) in situ. This reaction is known to enhance the efficiency of H_2_O_2_ utilization when coupled with monooxygenases or peroxidases (Robbins et al. 2018a). The high atom economy of FOx-catalyzed reactions and the straightforward separation of the byproduct, CO_2_, render it well suitable for H_2_O_2_-dependent biosynthetic reactions (Li et al. 2022). Furthermore, during the catalytic cycle, the flavin cofactor in FOx is reduced to hydroquinone, a form recently shown to be a highly controllable and effective radical source for light-driven enzyme-catalyzed reversible addition-fragmentation chain transfer (RAFT) polymerization. The high turnover rate of FOx holds promise for its extensive application in industrial biocatalysis (Heath and Turner 2022; Lao et al. 2022; Tao et al. 2024).

FOx exhibits distinct kinetic characteristics compared to its counterparts within the GMC superfamily (Heuts et al. 2009). Specifically, the turnover rate of FOx gradually increases over tens of hours following the purification (Doubayashi et al. 2019). Recent studies have reported that FOx contains a rare 8-formyl flavin adenine dinucleotide (8-formyl FAD) cofactor, instead of FAD found in other GMC superfamily members (Maeda et al. 2009b; Wongnate and Chaiyen 2013). This covalently modified cofactor is generated in situ from FAD through self-oxidation mechanism. Consequently, it is hypothesized that the oxidative maturation of the cofactor contributes to the progressive enhancement of the FOx turnover number (Robbins et al. 2017). Notably, FOx is not the only protein reported to contain 8-formyl FAD (Edmondson 1974). The heterodimeric human electron-transferring flavoprotein (hETF), isolated under pH 8.5, has shown a conversion of its cofactor from FAD to 8-formyl FAD, accompanied by 50% reduction in turnover number (Augustin et al. 2018). Additionally, site-directed mutagenesis studies of lactate oxidase (LOx) have demonstrated that the R286L-LOx variant contains 8-formyl flavin mononucleotide (8-formyl FMN), but this variant exhibits almost no activity (Yorita et al. 2000). Thus, among all known enzymes containing 8-formyl FAD, the 8-formylation modification appears to only enhance the catalytic ability of FOx. Redesigning the active sites of FAD-dependent enzymes to accommodate 8-formyl FAD presents an optimistic and potential strategy for enhancing the catalytic function. However, it is crucial to assess whether the microenvironment within enzyme’s active pocket can facilitate the formation of 8-formyl FAD, and equally important to ensure that 8-formyl FAD possesses the appropriate charge distribution and conformation to realize its catalytic ability. Therefore, elucidating the mechanism behind the formation and functional engagement of 8-formyl FAD in FOx will undoubtedly facilitate the development of flavoprotein oxidases with enhanced oxidative capability.

Extensive investigations using enzyme kinetics, site-directed mutagenesis, and spectroscopic analysis have sought to elucidate the formation mechanism of 8-formyl FAD cofactor in FOx and its catalytic mode (Robbins et al. 2017, 2018b; Willot et al. 2020). Despite these efforts, the precise mechanistic details are still not fully understood. A central debate focuses on the potential roles of specific amino acids in the FAD autoxidation reaction and their contributions to this process. A hypothesis suggests that the deprotonation of methyl group at the C8 atom of FAD by a general base facilitates the formation of a quinone-methide tautomeric intermediate. This intermediate is then considered to be stabilized through a covalent linkage formed by residue S94 at the C8 atom of 8-formyl FAD. The process culminates in the generation of 8-formyl FAD through a series of proton and electron transfer, where water molecules and general bases facilitate the proton transfer, and oxygen molecules act as electron acceptors (Augustin et al. 2018; Heuts et al. 2009). However, two critical questions remain unresolved in this model. Firstly, the identity of the general base remains unclear—is it the residue R87 or a hydroxide ion in solution? Notably, crystallographic analysis has shown that three residues, S94, R87 and L97, are positioned within 10.0 Å of the C8 atom (Maeda et al. 2010). Secondly, the mechanism by which the deprotonated state of S94 is achieved requires further clarification, as this state is necessary for covalent binding to FAD (Blay and Pei 2019).

An alternative reaction pathway for 8-formylation within the oxidation mechanism of riboflavin-5’-phosphate to 8-demethyl-8-aminoriboflavin-5’-phosphate (AFP) catalyzed by AFP synthase has been proposed (Konjik et al. 2017). In this model, the formylated flavin transiently appears as an intermediate, with hydroxide ions acting as general bases to deprotonate the methyl group at the C8 atom of flavin. Residues near the O4 atom are suggested to stabilize the quinone-methide tautomeric intermediate. Subsequent oxidation by oxygen molecules directly yields the 8-hydroperoxide anion of riboflavin-5’-phosphate, which ultimately converts into 8-formyl riboflavin-5’-phosphate in the presence of hydroxide ions and water at the active site. Although this alternative pathway offers a more streamlined reaction mode, it fails to address whether residues near the C8 atom are involved in the reaction process. Therefore, getting deeper insight into the 8-formyl FAD formation through a more rigorous analysis of the involved processes will improve the utilization of this cofactor and deepen our understanding of flavin cofactor modification.

In this study, we conducted a comprehensive investigation into the mechanism of FAD oxidation to 8-formyl FAD in FOx by integrating QM/MM umbrella sampling (US) simulation, molecular dynamics (MD) simulation, and site-directed mutagenesis. Specifically, our analysis meticulously evaluated the reaction coordinates (RCs) associated with the residues R87 and S94, which are directly involved in the oxidation process, as well as RCs for reactions not involving these residues directly. Additionally, we investigated the functional roles of R87 and S94 using site-directed mutagenesis and MD simulation. Our comprehensive analysis elucidated all intermediates in the oxidative maturation of 8-formyl FAD, quantified the free energy barrier for each reaction step, and identified the critical rate-limiting step in the pathway. Using the QM/MM US method, the essential role of 8-formyl FAD was elucidated in the catalytic functionality of FOx. Overall, our findings provide new insights into the formylation modification of FAD and enhance our understanding of flavin modification. These results also offer theoretical guidance for the development of modified flavin-dependent oxidases, which are crucial for biomanufacturing applications.

## Results

### The central role of 8-formyl FAD in FOx-mediated conversion of formate to CO_2_

**Fig. 1:**
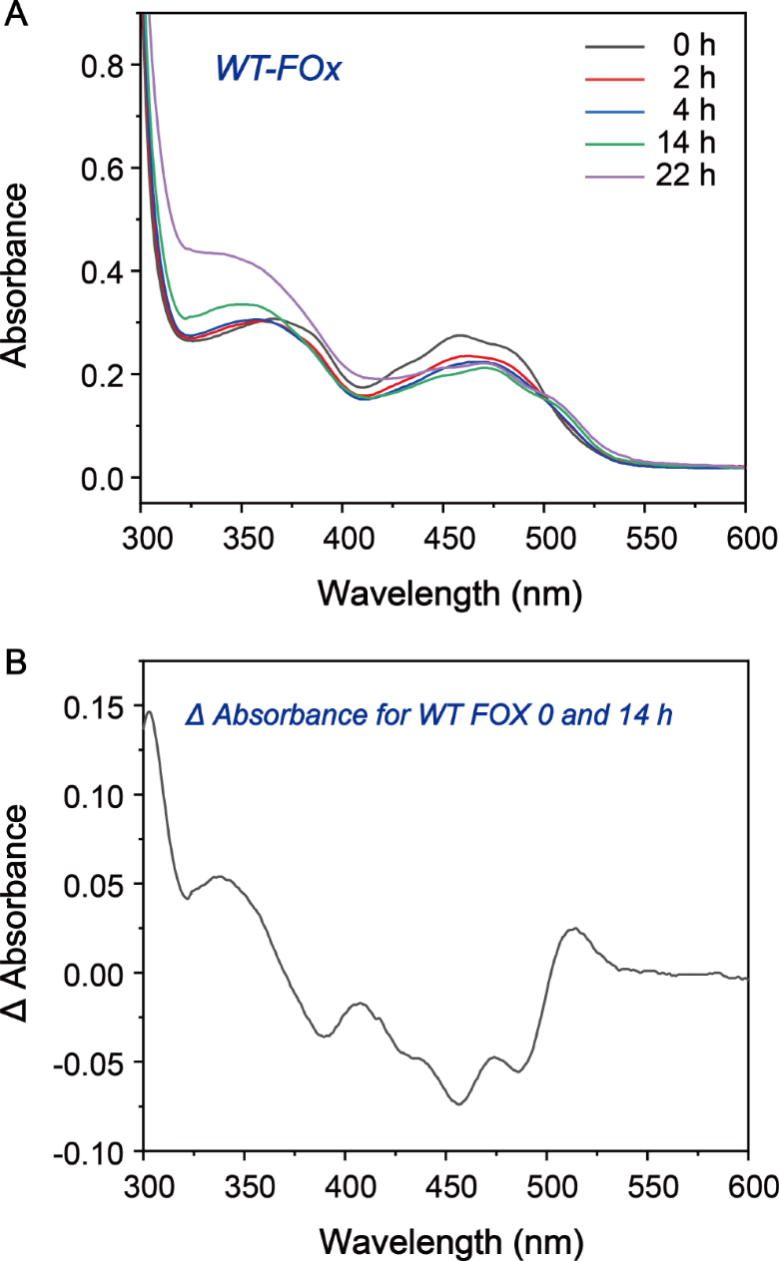
Time dependence of 8-formyl FAD formation in WT FOx. (**A**) UV-visible absorption spectra ranging from 300 to 600 nm were recorded at various time points: 0 h (black), 2 h (red), 4 h (blue), 14 h (green), and 22 h (purple) following cell lysis. (**B**) The changes in the absorption spectrum of WT FOx between the initial measurement at 0 h and after 14 h post-cell lysis

To assess whether FAD bound to FOx could self-catalytically convert to 8-formyl FAD in vitro, we monitored the UV-visible absorption spectra of the freshly purified enzyme over time (Fig. 1A). A comparison of the absorption spectrum of wild-type (WT) FOx after 14 h of incubation with that of fresh purification revealed a maximal increase of absorbance at 512 nm, and peaks at shorter wavelengths exhibited a blue shift (Fig. 1B). These results indicated the conversion of FAD to 8-formyl FAD within the active site of FOx without the addition of exogenous compounds. The formate oxidizing activity of WT FOx, as shown by the *k*_cat_ value, increased from 78 ± 5.2 to 102 ± 5.7 s^− 1^ over 14 h (Table S1). After approximately 24 h, the enzyme began to aggregate and precipitate, resulting in a marked decrease in formate oxidizing activity.

**Fig. 2:**
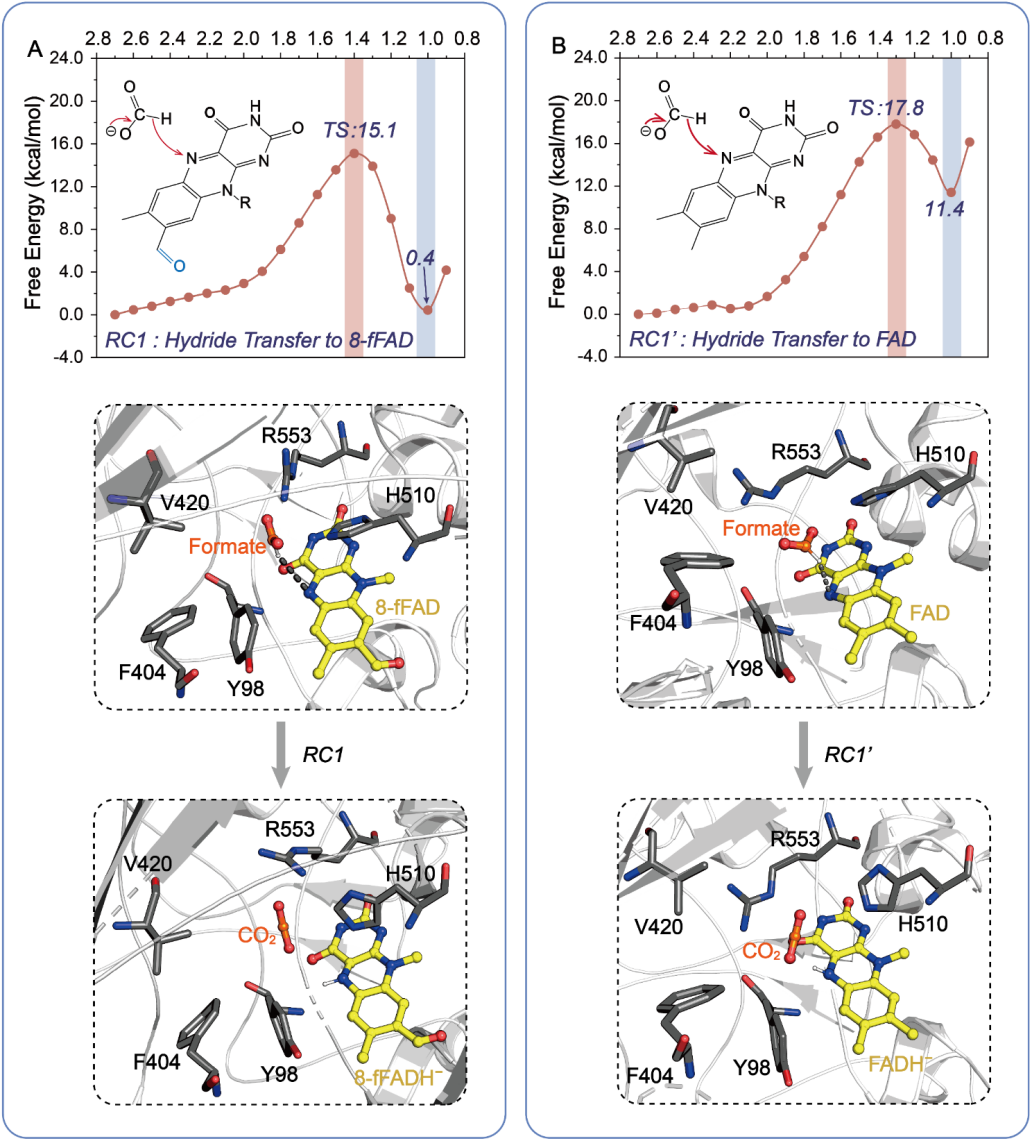
The free energy barriers associated with the hydride transfer from formate to 8-formyl FAD (**A**) and to FAD (**B**), reflecting the differences in the efficiency of formate oxidation catalyzed by FOx bound to 8-formyl FAD and FAD

To elucidate the differences in the efficiency of formate oxidation catalyzed by FOx bound to FAD and 8-formyl FAD, we subsequently performed QM/MM US simulation. We defined two reaction coordinates, **RC1** and **RC1’**, as the distances from the hydrogen atom of formate to the N5 atom of 8-formyl FAD and FAD cofactors, respectively (Table S2). The free energy profiles and the two-electron oxidation mechanism are depicted in Fig. 2. It was observed that the hydride transfer from formate to 8-formyl FAD surmounts an energy barrier of 15.1 kcal mol^− 1^, which is lower than the 17.8 kcal mol^− 1^ barrier for the FAD cofactor. Applying Eyring’s equation, and using the *k*_cat_ value for FOx with 8-formyl FAD bound (102 ± 5.7 s^− 1^), we estimated an activation energy of approximately 15.0 kcal mol^− 1^, closely matching the calculated energy barrier of 15.1 kcal mol^− 1^. Furthermore, the computed free energy for FOx with FAD bound (17.8 kcal mol^− 1^) generally coincides with the estimated energy (16.5 kcal mol^− 1^) derived from the *k*_cat_ value (11 ± 2.0 s^− 1^) (Robbins et al. 2017). Thus, the kinetic data align well with the computational energy values, demonstrating that FOx containing 8-formyl FAD is more active in the oxidation of formate than that containing FAD.

### Mechanism of self-catalytic conversion of FAD to 8-formyl FAD

**Fig. 3:**
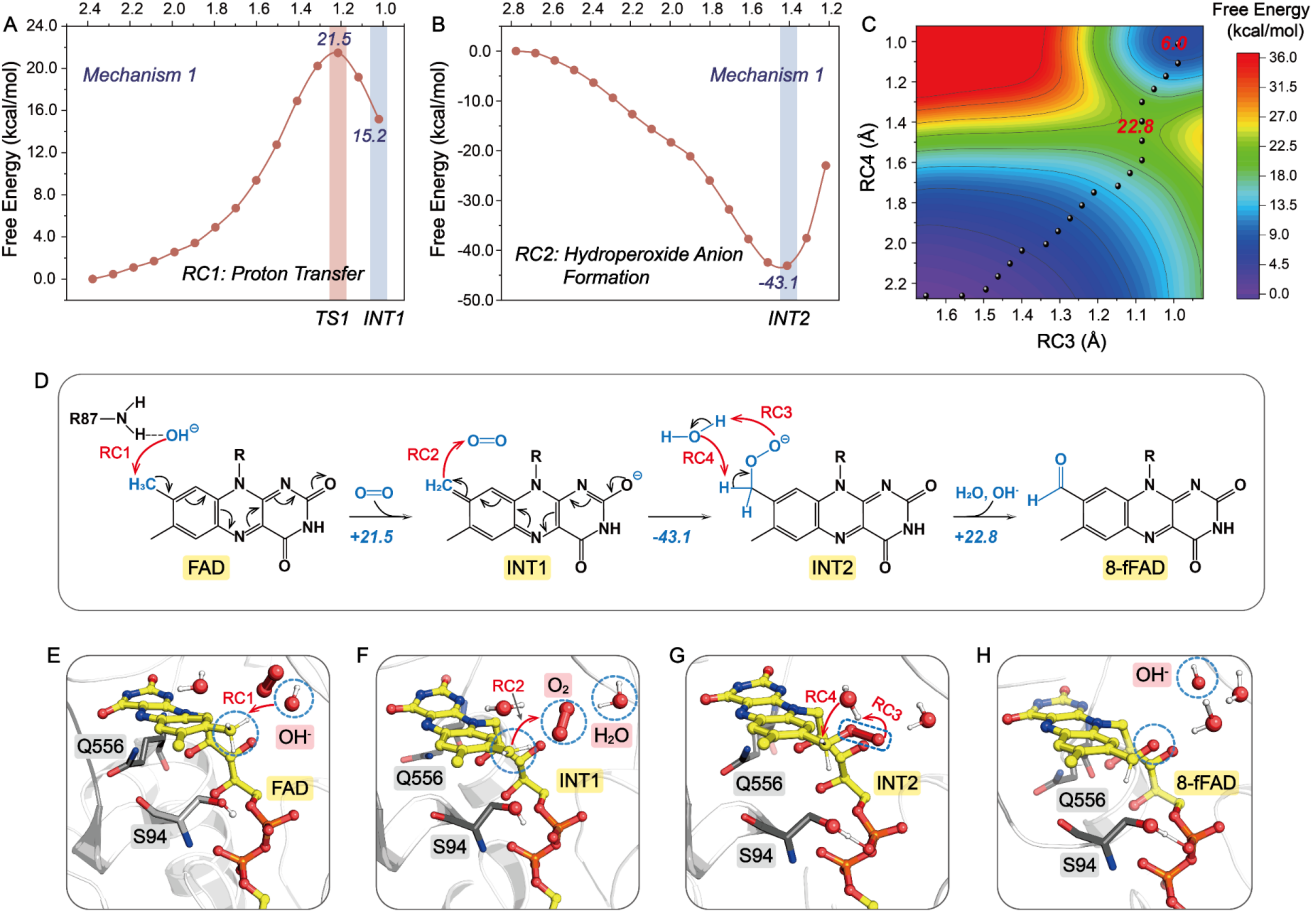
Free energy profiles for the deprotonation of the C8 methyl group on the isoalloxazine ring of FAD catalyzed by the catalytic OH^−^ group (**A**), the formation of the C8 methyl hydroperoxide anion (**B**), and the protonation of the resulting hydroperoxide anion to generate OHC-FAD and OH^−^ groups (**C**). (**D**) Mechanistic Scheme I for the self-catalytic conversion of FAD to 8-formyl FAD. (**E-H**) The structures of **FAD**, **INT1**, **INT2** and **8-fFAD** present the formation mechanism of 8-formyl FAD

Structural data and mutagenesis experiments on AFP synthase (RosB) have elucidated the roseoflavin biosynthesis pathway and proposed a corresponding oxidative reaction mechanism (Konjik et al. 2017). This mechanism reveals that the C8 methyl proton of riboflavin-5’-phosphate (RP) does not interact directly with any catalytic amino acids. Instead, it is directly transferred to a deprotonated water molecule within an anion hole. This catalytic water molecule, acting as a general base, deprotonates the C8 methyl group on the isoalloxazine ring of RP, leading to the formation of a quinone-methide tautomer intermediate. The intermediate is stabilized by hydrogen bond interactions between N1 and O2 of the isoalloxazine ring and the positively charged residues of RosB. An O_2_ molecule subsequently oxidizes the quinone-methide intermediate, resulting in a C8 methyl hydroperoxide anion that is protonated by a nearby H_2_O molecule. Then this C8 methyl hydroperoxide is dissociated to form OHC-RP and OH^−^. Building on the proposed oxidative reaction mechanism for RosB, we investigated the mechanistic pathways of self-catalytic conversion of FAD to 8-formyl FAD within the noncovalent FOx: FAD complex through DFT-based QM/MM US simulation. The corresponding reaction coordinates are depicted in Table S3. Initially, US simulation analyzed the deprotonation of the C8 methyl group on the isoalloxazine ring of FAD. In this **FAD**$\to$**INT1** simulation, the catalytic OH^−^ in the anion hole acts as a general base, abstracting the C8 methyl proton to form the reduced flavin species, overcoming an energy barrier of 21.5 kcal mol^− 1^ (Fig. 3A, D, and E). Following the formation of this intermediate (**INT1**), a nearby O_2_ molecule readily attacks the quinone-methide tautomer (**INT1**$\to$**INT2**) (Fig. 3B, D, and F). The resulting C8 methyl hydroperoxide anion is then protonated by a nearby water molecule, leading to the dissociation into OHC-FAD and OH^−^ across reaction coordinates **RC3** and **RC4** (**INT2**$\to$**8-fFAD**). These two sub-steps were characterized by a feasible energy barrier of 22.8 kcal mol^− 1^ (Fig. 3C, D, G, and H).

### Role of R87 in the mechanism of FAD conversion to 8-formyl FAD

**Fig. 4:**
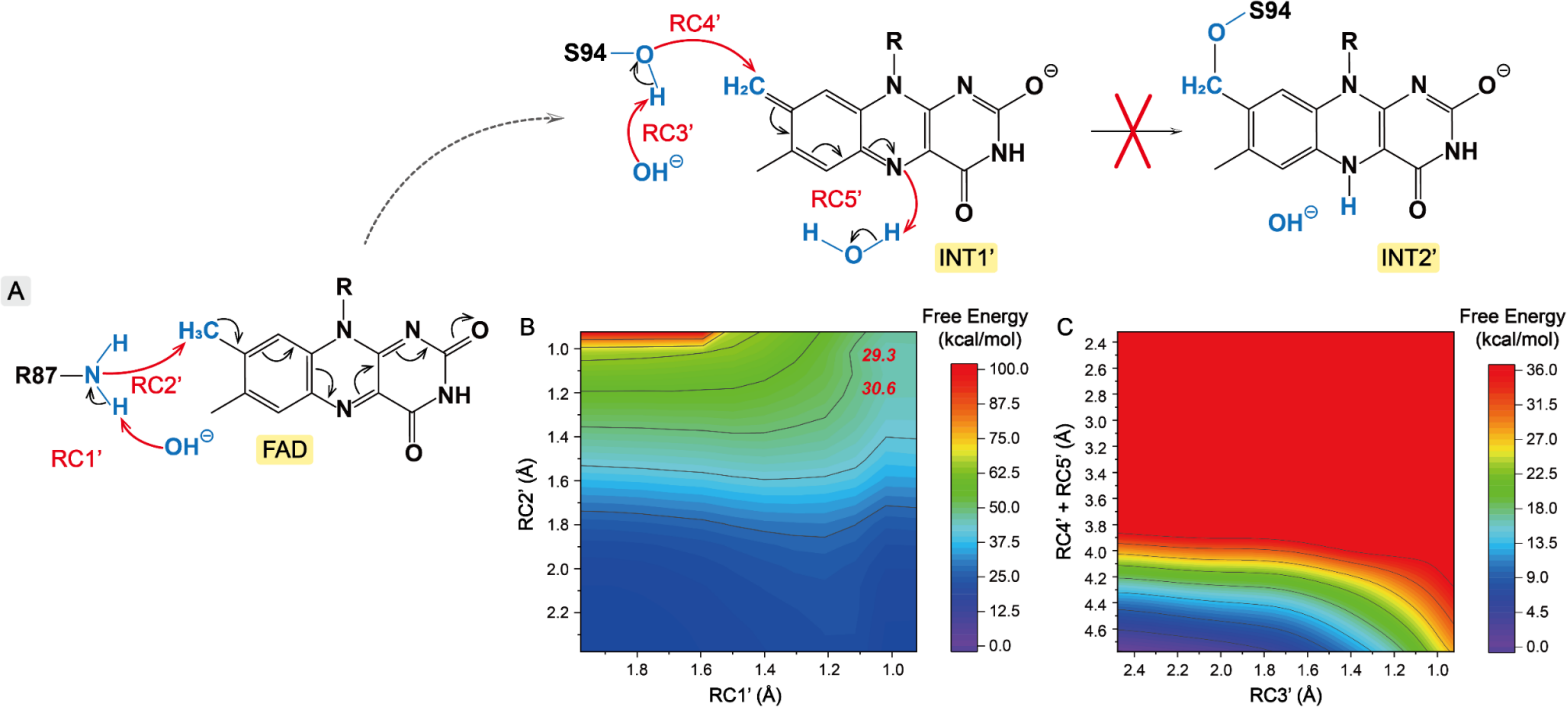
(**A**) Mechanistic Scheme II for the self-catalytic conversion of FAD to 8-formyl FAD. Free energy profiles for the deprotonation of C8 methyl group of FAD catalyzed by the catalytic R87 residue (**B**), and for the formation of the covalent intermediate resulting from the attack by S94 on the quinone-methide tautomer (**C**)

Analysis of the FOx catalytic site revealed that the arginine residue, R87, is located in van der Waals contact with the C8 methyl group on the isoalloxazine ring of FAD. Thus, we explored an alternative mechanism where the deprotonation of C8 methyl group occurs by transferring a proton to the deprotonated R87, facilitated by a nearby catalytic OH^−^ along reaction coordinates **RC1’** and **RC2’** (Fig. 4). In these coordinated sub-steps, the transition state (TS) is characterized by an energy of 30.6 kcal mol^− 1^ at distances of approximately 1.0 and 1.2 Å for the reaction coordinates of R87 and C8 methyl deprotonations, respectively. Notably, this TS energy is 9.0 kcal mol^− 1^ higher than that observed in the **FAD**$\to$**INT1** simulation, where R87 is not directly involved, suggesting that a hydroxide ion in the anion hole is more favorable as a general base to initiate the reaction.

**Fig. 5:**
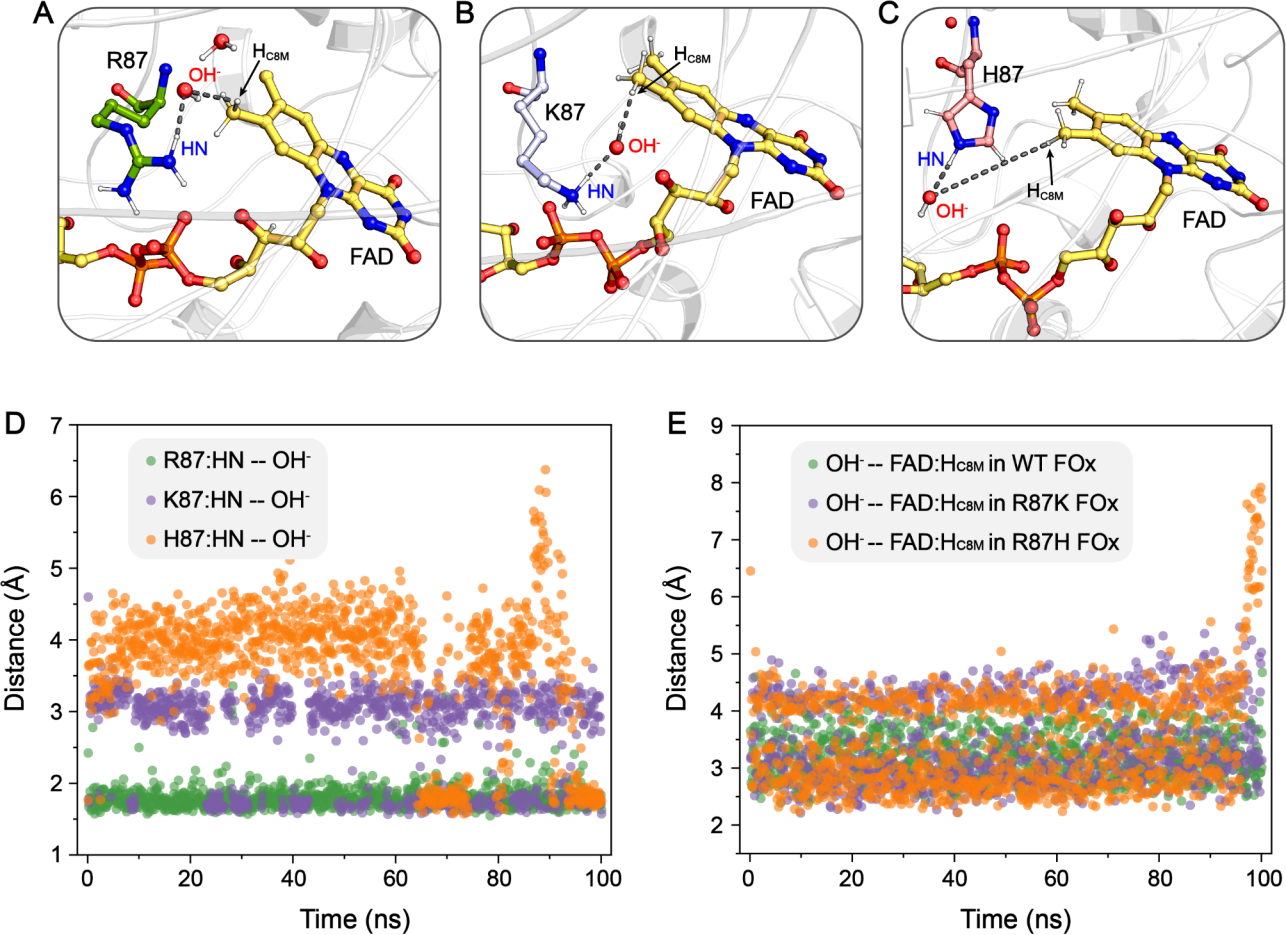
Crucial interactions facilitating the attack by OH^−^ in the anion hole on the C8 methyl group for WT FOx (**A**) and its mutants R87K (**B**) and R87H (**C**). The changes in the distances between R87/K87/H87:HN and OH^−^ (**D**), as well as between OH^−^ and FAD: H_C8 methyl_ (**E**) throughout the 100-ns MD simulation

The autocatalytic formation of 8-formyl FAD is dependent on R87, as evidenced by the R87A variant of FOx, which contained only FAD as indicated by a single species eluting at 4 min in the LC-MS chromatogram (Robbins et al. 2017). Consequently, further analysis was conducted to clarify R87’s role in the formation of 8-formyl FAD. The UV-visible absorption spectra of freshly purified R87H and R87K variant of FOx were monitored over time, revealing the presence of both FAD and 8-formyl FAD in the R87K variant, while only FAD was detected in the R87H variant (Fig. S1). Thus, the R87H mutation impairs the capacity for self-catalytic conversion of FAD to 8-formyl FAD. To elucidate the underlying molecular mechanism, molecular dynamics simulation was performed on the WT, R87K and R87H FOx systems. The MD-equilibrated structure of the WT system revealed a well-organized OH^−^ chain stabilized between the side chain of R87 and the C8 methyl group of FAD, as demonstrated by the two interaction distances R87:HN$\cdots$OH^−^ and OH^−^$\cdots$FAD: H_C8 methyl_, each less than ∼ 4.0 Å (Fig. 5A, D, and E). In the R87K system simulation, the distance of K87:HN$\cdots$OH^−^ increased by about 1.0 Å compared to the WT system, thereby reducing the likelihood of OH^−^ attacking the C8 methyl group (Fig. 5B, D, and E). Consequently, the efficiency of self-catalytic conversion of FAD to 8-formyl FAD in the R87K FOx is significantly slower than the autoxidation observed in WT FOx (Fig. S1). This decreased efficiency is also reflected in the reduced *k*_cat_ value for R87K FOx (76 ± 0.1 s^− 1^ at 14 h) compared to that of WT FOx (102 ± 5.7 s^−1^ at 14 h), as depicted in Table S4. The mutant R87H results in a shorter side chain of H87, which fails to properly position the OH^−^ group near the FAD: H_C8 methyl_ atom. This misalignment leads to a dramatic decrease in the concentration of hydroxyl radicals within the catalytic center, substantially inhibiting the deprotonation of C8 methyl group of FAD (Fig. 5C-E). Consequently, the formate oxidizing activity is nearly lost in the R87H FOx (Table S5). These findings suggest that R87 does not act as an active site base for proton deprotonation in the formation of the quinone-methide tautomer intermediate. Instead, R87 likely plays a critical role in the stabilization of general base OH^−^ group in the catalytic center during the formation of 8-formyl FAD.

### Role of S94 in the mechanism of FAD conversion to 8-formyl FAD

**Fig. 6:**
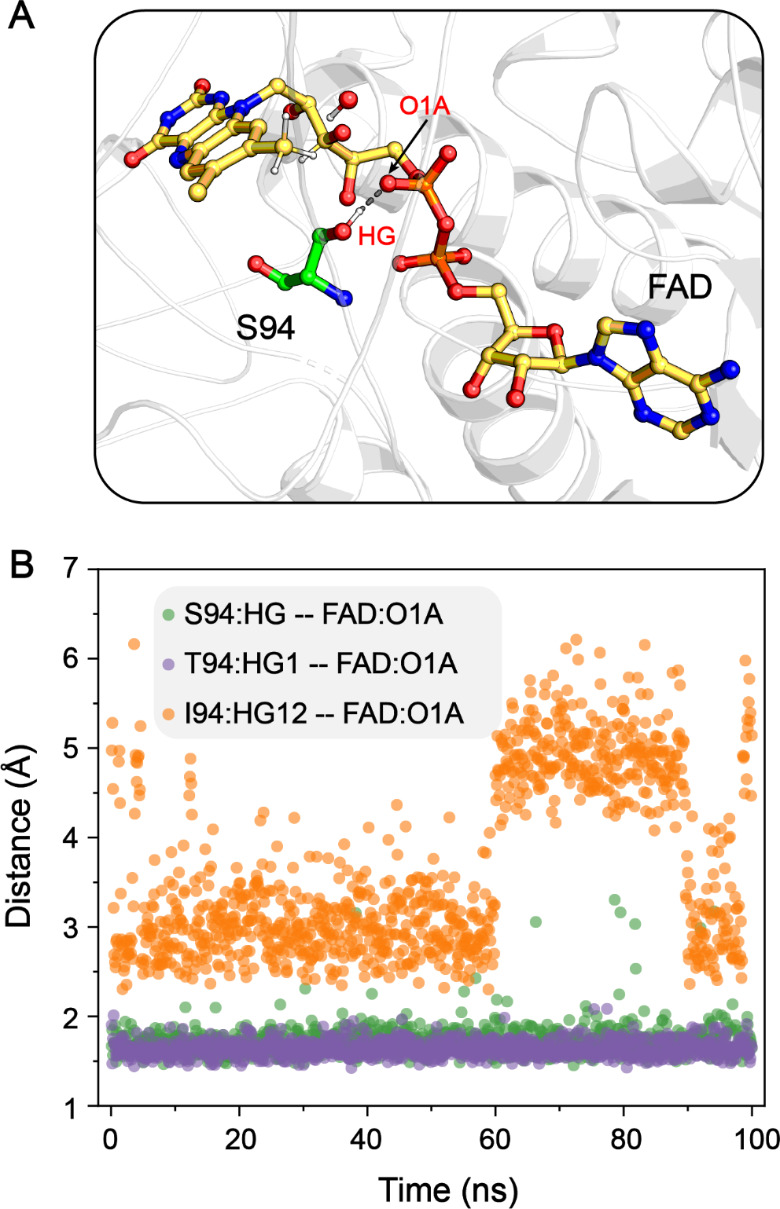
(**A**) Hydrogen-bonding interaction between the hydroxyl group of S94 and the oxygen atom of the phosphoryl moiety on FAD. (**B**) Changes in the distances between S94:HG and FAD: O1A, T94:HG1 and FAD: O1A, as well as I94:HG12 and FAD: O1A, observed over 100-ns MD simulation

Prior to the full reduction of the FAD intermediate, the quinone-methide tautomer may be stabilized by forming a covalent linkage with catalytic residues in FOx (Robbins et al. 2017). In this mechanism, a serine residue (S94) adjacent to the C8 region of the isoalloxazine ring is proposed to perform a nucleophilic attack on this quinone-methide tautomer. Then, we investigated the formation of the covalent intermediate by exploring **INT1’**$\to$**INT2’** process along the reaction coordinates **RC3’**, **RC4’**, and **RC5’**. The computed free energy barrier exceeded 30.0 kcal mol^− 1^, indicating that the stabilization through the covalent linkage between S94 and FAD is impossible (Fig. 4). Another mechanism stabilizing the intermediate involves a glutamine (Q556) near the N(1)C(2) = O(2) region of the isoalloxazine ring, which could stabilize the negative charge on the pyrimidine moiety of the intermediate. Additionally, a hydrogen bonding network between S94 and two phosphoryl groups of FAD might be essential for the stable FAD binding. To further investigate the role of S94 in 8-formyl FAD formation, we mutated S94 to isoleucine (I94), which has a similarly sized side chain, and purified the S94I variant under the same condition as WT FOx. The purified S94I variant protein, obtained from the elution with 200 mM imidazole, exhibited a colorless appearance. Characteristic absorption at 370 and 450 nm associated with the cofactor FAD was absent in this fraction (Fig. S2). Furthermore, the formate oxidase activity of the S94I variant was significantly reduced compared to WT FOx (Table S6). These observations suggest that the reduced FAD binding capability of S94I variant may be caused by the absence of a hydroxyl group at I94. We then mutated S94 to threonine (T94), remaining the hydroxyl group on the side chain, and found that the S94T variant does bind flavins, albeit partially. The purified protein solution was yellow in color, with characteristic UV-visible absorption peaks at 370 and 450 nm (Fig. S3). To further study the corresponding mechanism, molecular dynamics simulation was performed for the WT, S94T and S94I FOx systems. In the simulation of WT and S94T FOx systems, the distances of S94: HG$\cdots$FAD: O1A and T94: HG1$\cdots$FAD: O1A were centered at ∼ 1.8 Å, indicating the stable binding of FAD to the catalytic center through hydrogen-bonding interaction between the hydroxyl group of S94/T94 and the oxygen atom of the phosphoryl moiety on FAD (Fig. 6). In comparison to the WT and S94T systems, a higher degree of distance fluctuation was detected between I94:HG12 and FAD: O1A atoms in the S94I system (Fig. 6). These experimental and computational results demonstrated that the residues with hydroxyl side chains, such as S94 and T94, facilitate the binding of flavins into the catalytic center.

**Fig. 7:**
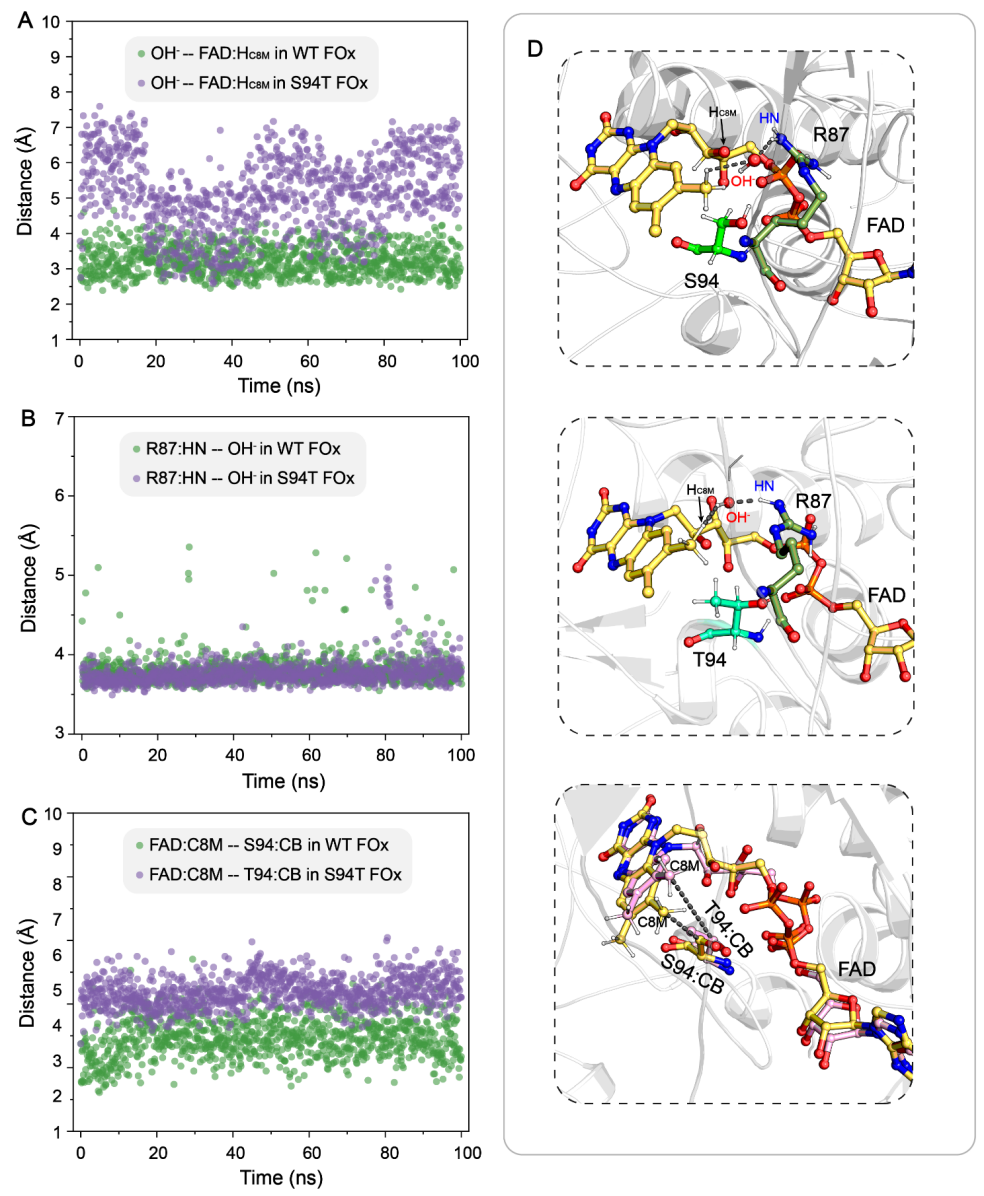
(**A**) Changes in the attacking distance between the catalytic OH^−^ and the H_C8 methyl group_ atom of FAD in both WT and S94T FOx. (**B**) Changes in the hydrogen bonding distance between R87 and the catalytic OH^−^ in both WT and S94T FOx. (**C**) Changes in the distance between the C8 methyl group of FAD and the CB atom of S94/T94 in both WT and S94T FOx. (**D**) Visualization of key interaction in both WT and S94T FOx

Compared to the high activity of WT FOx (*k*_cat_ of 102 ± 5.7 s^− 1^), the S94T variant exhibited a significantly reduced *k*_cat_ value of 4 ± 0.1 s^− 1^ at atmospheric oxygen concentration after 14 h (Tables S1 and S7), indicating a substantial decrease in formate oxidase activity. To further elucidate the critical role of S94 residue in maintaining formate oxidase activity, we monitored the orientation of the nucleophilic OH^−^ group during MD simulation. Notably, the attacking distance between the catalytic OH^−^ and the H_C8 methyl group_ atom of FAD in S94T FOx was significantly longer than that in WT FOx, resulting in decreased efficiency of proton transfer via the nucleophilic attack on the C8 methyl group of FAD (Fig. 7A). Additionally, we observed that the R87 residue, which plays a crucial role in stabilizing the general base of OH^−^ group, exhibited minimal differences in hydrogen bonding with OH^−^ between the WT and S94T FOx systems (Fig. 7B). Interestingly, the distance between the C8 methyl group of FAD and the CB atom of T94 was 1.5 Å longer than the corresponding distance in WT FOx system (Fig. 7C). This additional methyl side chain in T94 introduces steric hindrance in the active center, causing the isoalloxazine ring of FAD to deviate slightly from the stably bound catalytic OH^−^ group. The deviation slows down the initial step of proton transfer in the formation of 8-formyl FAD by increasing the distance required for the nucleophilic attack. Overall, we can conclude that the hydroxyl group of S94/T94 residues facilitates the FAD binding in the active center; however, the methyl side chain of T94 spatially impedes the proton transfer from the C8 methyl group of FAD to a general base, thus affecting the catalytic efficiency.

## Discussion

Flavoprotein enzymes are extensively utilized in enzyme-catalyzed chemical synthesis due to the critical role of flavin cofactors in electron and proton transfer (Linke et al. 2023; Sun et al. 2022). The cofactor in FOx differs from those in other members of the GMC superfamily. In the presence of oxygen, FOx’s FAD cofactor spontaneously oxidizes to 8-formyl FAD (Doubayashi et al. 2011). Although this oxidation process is slow, it results in a notable increase in the enzyme’s *k*_cat_ value during the gradual oxidation of FAD to 8-formyl FAD. FOx is distinctive not only because it contains the rare 8-formyl FAD but also as the sole enzyme identified where 8-formyl FAD could enhance the catalytic activity (Robbins et al. 2017). For enzyme molecules, there are numerous factors which can enhance the catalytic efficiency, including the alteration in the microenvironment’s charge or pH, the reduction in the transition state energy, and the improved induced-fit ability between the active site and substrates. However, how 8-formyl FAD in FOx enhances the catalytic efficiency has not been sufficiently elucidated. In our study, we explored why 8-formyl FAD, rather than FAD, executes the catalytic function of FOx. Using QM/MM US simulation, we calculated the energy barrier for the deprotonation of formate substrate and found that 8-formyl FAD lowers this barrier by 2.7 kcal mol^− 1^ compared to FAD. The finding suggests that 8-formyl FAD within the active site of FOx, exhibits a stronger oxidizing capability and more readily accepts the electrons from formate.

However, when flavin cofactors are free in the environment, it is difficult to produce functional modifications. This highlights the importance of the enzyme’s active site environment for the maturation and functional expression of flavin modifications (Leys and Scrutton 2016). Within the environment, critical non-covalent interactions occur between the cofactor and the side chains or backbones of amino acids, as well as the interactions with molecular oxygen. In previous research on formate oxidase, it was hypothesized that a general base could deprotonate the C8 methyl group on FAD during the formation of 8-formyl FAD. Nevertheless, the specific identity of this base remained unresolved. Meanwhile, it was suggested that the active site might interact covalently with this deprotonated methyl group, thereby stabilizing the intermediate, though this residue also remained unidentified. Clearly, these investigations did not comprehensively reveal the formation mechanism of 8-formyl FAD in FOx. Thus, our study elucidated the mechanism of each reaction step within the active site of FOx, specifically identifying the general base responsible for deprotonating the C8 methyl group as a catalytic hydroxide ion (OH^−^) situated near the active center. Subsequently, an adjacent O_2_ molecule attacks the quinone-methide tautomer, further stabilizing the deprotonated FAD intermediate.

Moreover, through detailed examination of the crystal structure, we identified two polar amino acid residues, specifically R87 and S94, located within 5 Å of the 8-formyl FAD’s C8 methyl group. These polar residues generally play crucial roles in the catalytic function of enzymes, with arginine involved in acid-base catalysis and serine in proton transfer. We hypothesized that these residues probably played either a direct or indirect role in the catalytic cycle. Our results indicated that R87 could stabilize the basic environment around the isoalloxazine ring, rather than directly participate in proton transfer. S94 was essential for the hydrogen-bonding network that stabilized the cofactor, and the mutation of S94 to a non-hydroxyl residue prevented the cofactor binding. Additionally, we found that the oxidative maturation of the cofactor was facilitated by Q556, which stabilized the negative charge of intermediates.

The enhanced catalytic properties of 8-formyl FAD could lead to the development of new biocatalysts with superior redox characteristics. For the application in other flavoenzymes, it is essential to replace residues around the C8 methyl group with suitable basic amino acids and ensure the presence of amino acids that can stabilize negatively charged intermediates near the isoalloxazine ring. Furthermore, 8-formyl FAD exhibits the absorption peaks at longer wavelengths, indicating that it requires lower excitation compared to FAD. When flavoenzymes dependent on 8-formyl FAD are used instead of those relying on FAD in photoenzymatic systems, they can be activated with shorter-wavelength blue light. This approach not only reduces the photodamage to proteins but also enhances the long-term viability of photoenzymatic systems. Consequently, it is a viable strategy to substitute natural FAD with 8-formyl FAD in photoenzymatic reactions.

## Conclusions

In this study, we systematically explored the specific reaction mechanism involved in the self-conversion of FAD to 8-formyl FAD. This process involves several steps: the deprotonation of the C8 methyl group on the isoalloxazine ring of FAD (**FAD**$\to$**INT1**), the formation of the C8 methyl hydroperoxide anion (**INT1**$\to$**INT2**), and the dissociation into OHC-FAD and OH^−^ groups (**INT2**$\to$**8-fFAD**). In the **FAD**$\to$**INT1** reaction, a catalytic water molecule serves as a general base to deprotonate the C8 methyl group of FAD, forming a quinone-methide tautomer intermediate. The proximal R87 residue stabilizes the OH^−^ group, thereby facilitating the necessary proton transfer. Subsequently, the intermediate is further stabilized by a hydrogen bonding network involving S94 and two phosphoryl groups of FAD. Following this stabilization, a nearby O_2_ molecule quickly attacks the quinone-methide tautomer (**INT1**$\to$**INT2**). The resulting C8 methyl hydroperoxide anion is then protonated by a nearby water molecule, leading to its dissociation into OHC-FAD and OH^−^ groups (**INT2**$\to$**8-fFAD**). Our results reveal that although residues R87 and S94 are not directly involved in the chemical reactions, the autocatalytic formation of 8-formyl FAD depends on these residues due to the noncovalent interactions they facilitate, which stabilize the nucleophilic OH^−^ group and the quinone-methide tautomer intermediate.

## Materials and methods

### Materials

*E. coli* Trans5α and *E. coli* BL21(DE3) strains, along with *ProteinRuler*^®^ IV, were purchased from TransGen Biotech (Beijing, China). The QuickMutation Site-Directed Mutagenesis Kit was purchased from Beyotime Biotechnology (Shanghai, China). The Plasmid Mini Kit was purchased from Omega (Guangzhou, China). The BCA Protein Quantification Kit was purchased from Sparkjade Biotechnology (Shandong, China). The One-Step PAGE Gel Fast Preparation Kit was purchased from Vazyme Biotech (Nanjing, China). The Ni-Sepharose 6FF column was purchased from Solarbio Science & Technology (Beijing, China). Sodium formate and sodium acetate were purchased from Yuanye Bio-Technology (Shanghai, China). 2,2′-Azino-bis(3-ethylbenzothiazoline-6-sulphonic acid) diammonium salt (ABTS) was purchased from J&K Scientific (Beijing, China). Horseradish peroxidase was purchased from BioDee Biotechnology (Beijing, China). Glycine, yeast extract, isopropyl β-D-thiogalactopyranoside (IPTG), kanamycin and imidazole were purchased from Gentihold Biotechnology (Beijing, China). Dipotassium hydrogen phosphate was purchased from Guangfu Technology Development (Tianjin, China), and potassium dihydrogen phosphate was purchased from Chemical Industry Group (Beijing, China). Agarose was purchased from Biowest (Shanghai, China).

### Cloning, expression and purification of FOx

The DNA sequence encoding FOx was synthesized by Hanbio Biotechnology (Shanghai, China) and subsequently cloned into the *Nde*I-*Hind*III restriction sites of pET28a(+) expression vector. The recombinant plasmid was transformed into *E. coli* Trans5α for sequence verification. After confirming the insertion of recombinant FOx gene, pET28a(+)-FOx was transformed into *E. coli* BL21(DE3) expression host. The *E. coli* BL21(DE3) cells were cultured at 37 ℃ with shaking at 180 rpm in LB medium supplemented with 50 µg/mL kanamycin. The expression was induced with 0.1 mM IPTG at an OD_600_ of 0.6–0.8, and the culture was continued at 20 ℃ for 7 h under the same shaking conditions. The cells were harvested by centrifugation and frozen at -20 ℃. For the purification of FOx, the cells were resuspended in 50 mM phosphate-buffered saline (PBS, pH 7.5) and disrupted by sonication. The cell lysate was clarified by centrifugation, and the supernatant was applied to a Ni-Sepharose 6FF column pre-equilibrated with 50 mM PBS (pH 7.5). Bound proteins were eluted with the same buffer containing 200 mM imidazole, and the protein concentration was determined using BCA Protein Quantification Kit. All the procedures were performed under light-protected conditions, either at 4 ℃ or on ice.

### Construction of variant proteins

The R87H, R87K, S94I and S94T FOx variants were engineered using the QuickMutation Site-Directed Mutagenesis Kit, with primers listed in Table S8. All mutated plasmids were confirmed by DNA sequencing analysis performed by Comate Bioscience Co. (Changchun, China), and those with the correct sequences were transformed into *E. coli* BL21(DE3) competent cells and stored at -80 ℃. Each variant was purified following the protocol as described in the section “Cloning, expression and purification of FOx”.

### Spectrum measurement

The UV-visible absorption spectra of purified FOx wild type and its variants were recorded at 4 ℃ after the incubation for 0, 2, 4, 14, 22, and 36 h using Shimadzu UV-2700 spectrophotometer (Shanghai, China). Measurements were conducted in a 1-cm quartz cuvette at a low scanning speed.

### FOx activity assay

The activity of FOx was determined using the horseradish peroxidase-based ABTS method, with sodium formate and ABTS as substrates. The reaction mixture containing ABTS (5 mM), horseradish peroxidase (10 U) and varying concentrations of sodium formate (0-400 mM), was prepared in 50 mM sodium acetate buffer (pH 5.0). 1 mL total volume of the mixture was used for each assay. Reactions were performed at 25 ℃, and changes in the absorbance at 420 nm were measured over the first 30 s following the initiation of the reaction at 0, 14, 24 and 36 h. The molar extinction coefficient for ABTS at 420 nm is 36,000 M^− 1^cm^− 1^. The reaction rates and kinetic parameters for FOx under different concentrations of sodium formate were calculated using the Michaelis-Menten equation and turnover number calculation equation (Srinivasan, 2022).

1 $${V}_{0} = \frac{{V}_{\text{m}\text{a}\text{x}}\left[S\right]}{{K}_{\text{m}} + \left[S\right]}$$

2 $${k}_{\text{c}\text{a}\text{t}}=\frac{{V}_{\text{m}\text{a}\text{x}}}{\left[E\right]}$$

where ${V}_{0}$ is the initial reaction rate, ${K}_{\text{m}}$ is the Michaelis constant, ${V}_{\text{m}\text{a}\text{x}}$ is the maximum reaction rate, $\left[S\right]$ is the substrate concentration, ${k}_{\text{c}\text{a}\text{t}}$ is the turnover number, and $\left[E\right]$ is the concentration of FOx enzyme.

### Calculation details of MD simulation

To elucidate the role of specific residues in the catalytic mechanism of FOx, a series of model systems were utilized, including WT FOx and its mutants R87K, R87H, S94I and S94T (Table S9). The initial geometries for the simulation were derived by modifying the crystal structure of the FOx monomer (PDB ID: 3Q9T) (Doubayashi et al. 2011). All water molecules in the original crystal structure were removed, and hydrogen atoms were added to the 8-formyl FAD cofactor. These variants were generated using the Mutagenesis tool in PyMOL software.

The MD simulation of FOx systems was conducted using the GPU-accelerated version of AMBER 2020 (Götz et al. 2012). The force field parameters for FAD, 8-formyl FAD, formate, oxygen, and hydroxide ions were generated using the Generalized Amber Force Field (GAFF) (Özpınar et al. 2010). Atom charges for these molecules were obtained through RESP charge fitting, based on electrostatic potentials calculated at the B3LYP/6-311G(d, p) level of theory (Scott and Radom 1996). Standard residues were modeled using the Amber ff19SB force field (Tian et al. 2020). Hydrogens were added to model structures using the LEaP module of AMBER, and each complex was solvated in a periodic boundary box filled with TIP3P water, neutralized with Na^+^ counterions (Mark and Nilsson 2001). The minimum distance between the enzyme surface and the edge of water box was set to 10.0 Å. All bonds involving hydrogen atoms were constrained using the SHAKE algorithm (Kräutler et al. 2001). Long-range electrostatics were managed with the particle mesh Ewald (PME) algorithm (Darden et al. 1993), with Lennard-Jones and electrostatic interaction cutoffs set at 9.0 Å. The system’s energy minimization with the steepest descent algorithm and conjugate gradient algorithm was executed to eliminate atomic collisions in the initial structure. Subsequently, the systems were gradually heated from 0 K to 303 K in an NVT ensemble using Berendsen temperature coupling (Berendsen et al. 1984), and then equilibrated in an NPT ensemble employing the Parrinello-Rahman pressure coupling (Berendsen et al. 1984). Production MD simulations was conducted for 100 ns under these conditions. The MD simulation trajectories were visualized and analyzed using the CPPTRAJ module (Roe and Cheatham 2013) and VMD software (Humphrey et al. 1996).

### Calculation details of QM/MM US simulation

To compare the catalytic activities of 8-formyl FAD and FAD cofactors, two additional systems were derived from the original WT FOx model. In the first system, formate was docked into the active site of WT FOx bound to 8-formyl FAD. In the second system, the cofactor was converted to FAD while retaining the configuration established in the first system. Additionally, to evaluate the feasibility of two proposed mechanistic schemes for the formation of 8-formyl FAD, corresponding models were developed based on the WT FOx system. For mechanistic Scheme I, as illustrated in Fig. 3, the cofactor was configured as FAD. To support the multi-step proton transfer proposed in Scheme І, four hydroxide ions were placed proximal to the C8 atom of FAD, alongside two oxygen molecules and four water molecules near the N5 atom. For mechanistic Scheme II, depicted in Fig. 4, a model was constructed with a water molecule, an oxygen molecule, and a hydroxide ion situated near the C8 atom.

The QM/MM US simulation (Kästner et al. 2006) was performed using the sander module implemented in Amber 20 (Götz et al. 2012). In evaluating the differential catalytic capabilities of 8-formyl FAD versus FAD, the quantum mechanics (QM) component of each system included the formate substrate, sidechain residues Y99, E394, F405, V421, F512, H513, R556 and the cofactor FAD/8-formyl FAD, excluding the phosphate group. For the analysis of mechanistic Scheme I, which is related to the formation mechanism of 8-formyl FAD, the QM component included sidechain residues S94, L97, N98, Y99, T101, R556, N558, along with a water molecule, an oxygen molecule, a hydroxide ion, and the cofactor FAD, also excluding the phosphate group. Correspondingly, in the calculation for mechanistic Scheme II, the QM component comprised sidechain residues R87, S94, L97, N98, Y99, T101, R556, N558, four water molecules, two oxygen molecules, four hydroxide ions, and the cofactor FAD without the phosphate group. Each QM/MM US simulation employed the second-order density functional tight binding level (DFTB2) theory (Seabra et al. 2007; Elstner et al. 1998; Niehaus et al. 2001) to characterize the QM component. The interface between the QM and molecular mechanics (MM) components was treated with hydrogen atoms to ensure smooth boundary conditions. The remainder of the FOx structure was assigned to the MM component and described using the Amber ff19SB force field (Tian et al. 2020). Long-range electrostatic interactions between QM and MM parts, as well as within the QM part, were computed using the particle mesh Ewald (PME) algorithm (Darden et al. 1993).

The QM/MM US simulation was employed to explore postulated reaction pathways and calculate the reaction free energy profile (FEP) (De Vivo et al. 2008; Rosta et al. 2011). The initial structure for each QM/MM US simulation was derived from a frame captured during MD simulation. The sampling windows along the reaction coordinate (RC) path were evenly spaced at 0.1 Å intervals, and each window was subjected to a harmonic potential of 200.0 kcal mol^− 1^. The first structure of each RC represented the structure in the last window of the preceding RC. Results from the US simulation were analyzed using the weighted histogram analysis method (WHAM) to obtain unbiased free energy profiles (Kumar et al. 1992). Additionally, minimum energy paths were investigated using the minimum energy path surface analysis (MEPSA) program (Marcos-Alcalde et al. 2015). Distances between atoms and representative snapshots from these analyses were visualized using the VMD (Humphrey et al. 1996) and PyMOL software.

### Electronic supplementary material

Below is the link to the electronic supplementary material.

Supplementary Material 1

Supplementary Material 2

## Data Availability

Data will be made available on reasonable request.
