# Supplementary material for: Mechanistic insights into the conversion of flavin adenine dinucleotide (FAD) to 8-formyl FAD in formate oxidase: a combined experimental and in-silico study
Source: Bioresour Bioprocess. 2024 Jul 10;11(1):67. doi: 10.1186/s40643-024-00782-4 (PMC11236828; doi:10.1186/s40643-024-00782-4)
Supplement: Supplementary file 1 — Supplementary Material 1 [file 40643_2024_782_MOESM1_ESM.docx]

**Supporting Information**

Mechanistic insights into the conversion of flavin adenine dinucleotide (FAD) to 8‑formyl FAD in formate oxidase: A combined experimental and in-silico study

Kai Wen ^a,#^, Sirui Wang ^a,#^, Yixin Sun ^a^, Mengsong Wang ^a^, Yingjiu Zhang ^a,^*, Jingxuan Zhu ^a,^*, Quanshun Li ^a,b,^*

*^a^Key Laboratory for Molecular Enzymology and Engineering of Ministry of Education, School of Life Sciences, Jilin University, Changchun 130012, China*

*^b^Center for Supramolecular Chemical Biology, Jilin University, Changchun 130012, China*

*Corresponding authors.

Tel. and Fax: +86-431-85155200.

E-mail: [yingjiu@jlu.edu.cn](mailto:yingjiu@jlu.edu.cn) (Y. Zhang); [zhujx@jlu.edu.cn](mailto:zhujx@jlu.edu.cn) (J. Zhu); [quanshun@jlu.edu.cn](mailto:quanshun@jlu.edu.cn) (Q. Li).

^#^These authors contributed equally to this work.


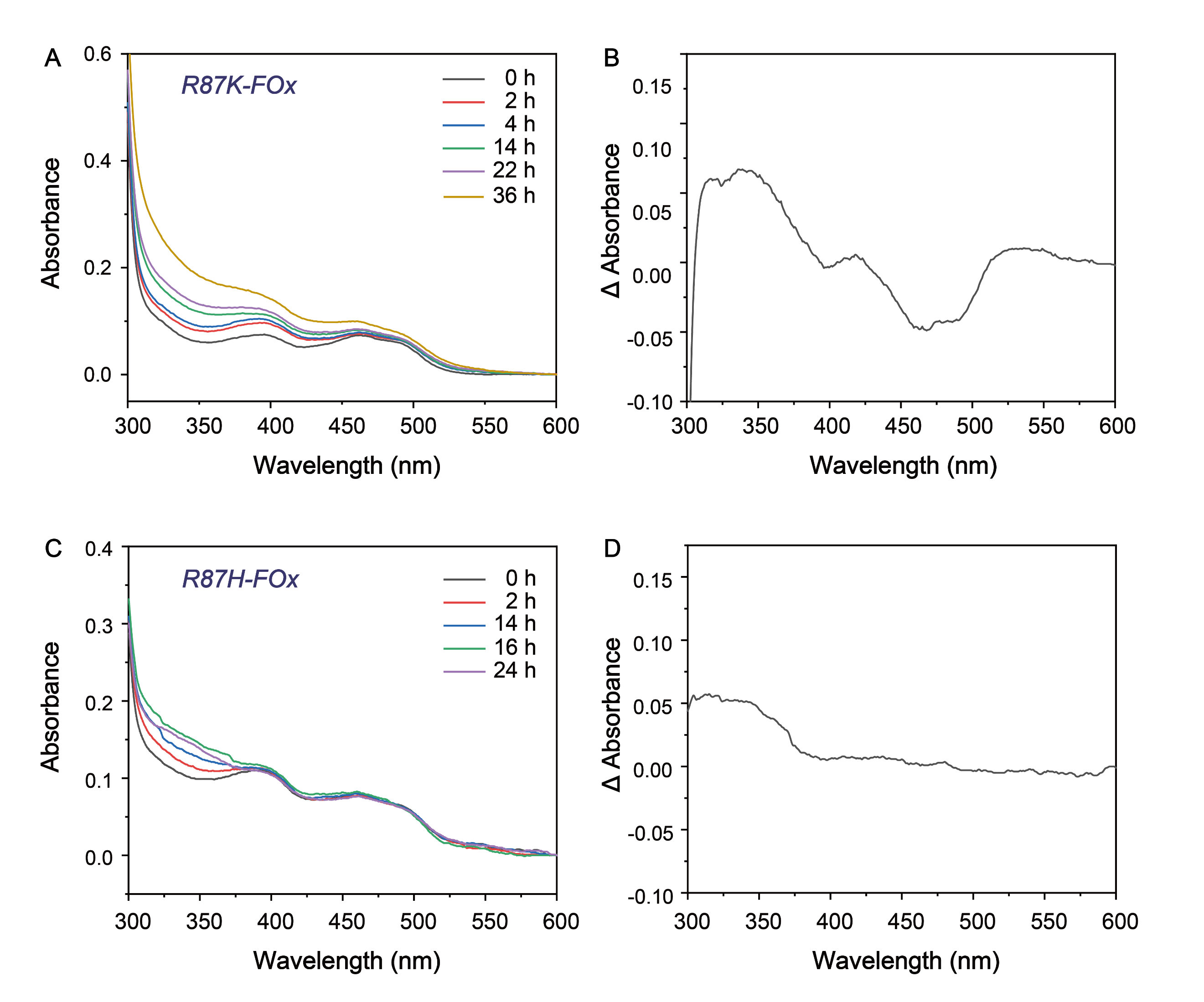


**Fig. S1.** Time dependence of 8-formyl FAD formation in FOx variants. (**A**) UV-visible absorption spectra of R87K FOx ranging from 300 to 600 nm were recorded at various time points: 0 h (black), 2 h (red), 4 h (blue), 14 h (green), 22 h (purple), and 36 h (yellow) following cell lysis. (**B**) The changes in the absorption spectrum of R87K FOx between the initial measurement at 0 h and after 36 h post-cell lysis. (**C**) UV-visible absorption spectra of R87H FOx ranging from 300 to 600 nm were recorded at various time points: 0 h (black), 2 h (red), 14 h (blue), 16 h (green), and 24 h (purple) following cell lysis. (**D**) The changes in the absorption spectrum of R87H FOx between the initial measurement at 0 h and after 14 h post-cell lysis.

**
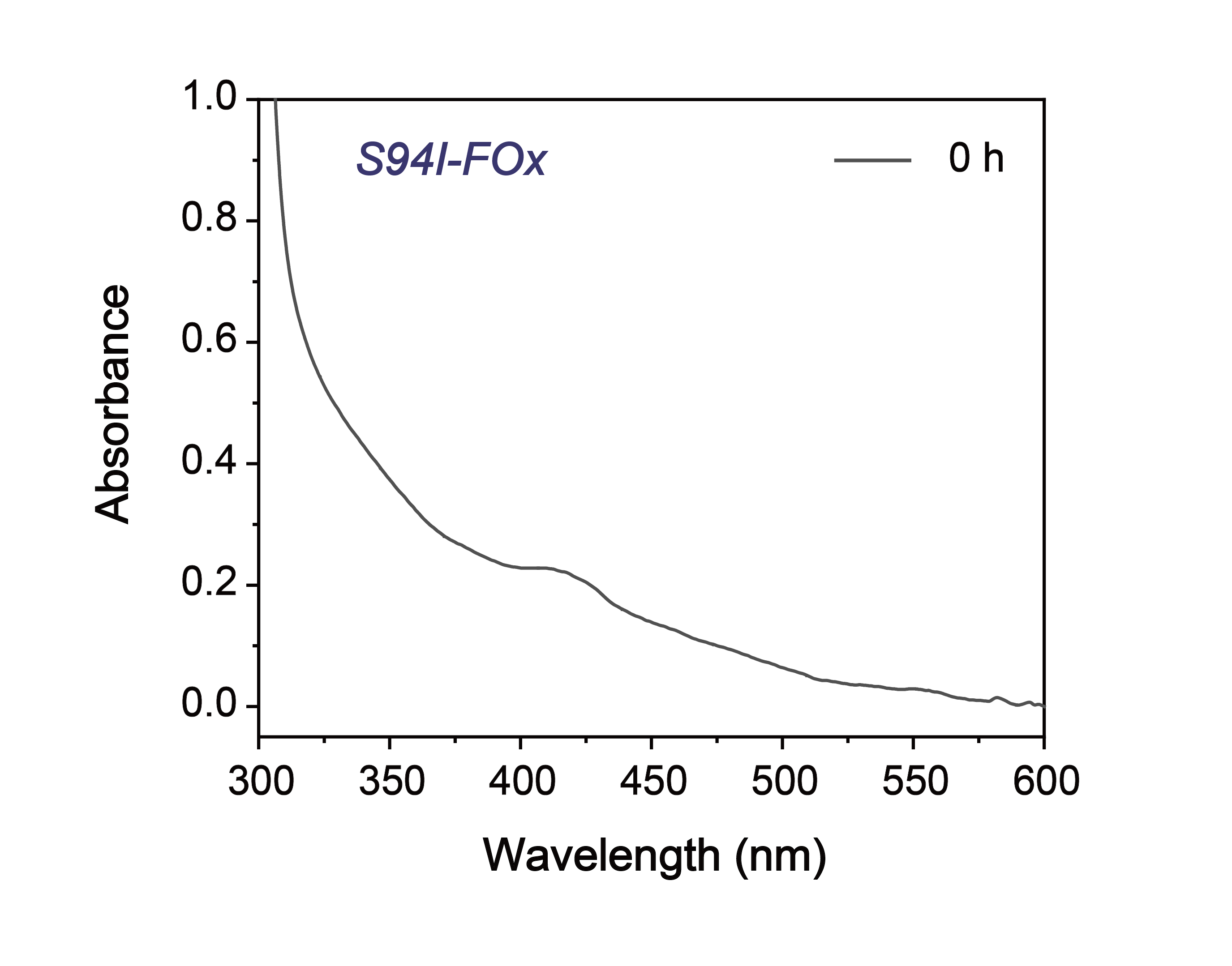
**

**Fig. S2.** UV-visible absorption spectrum of S94I FOx.

**
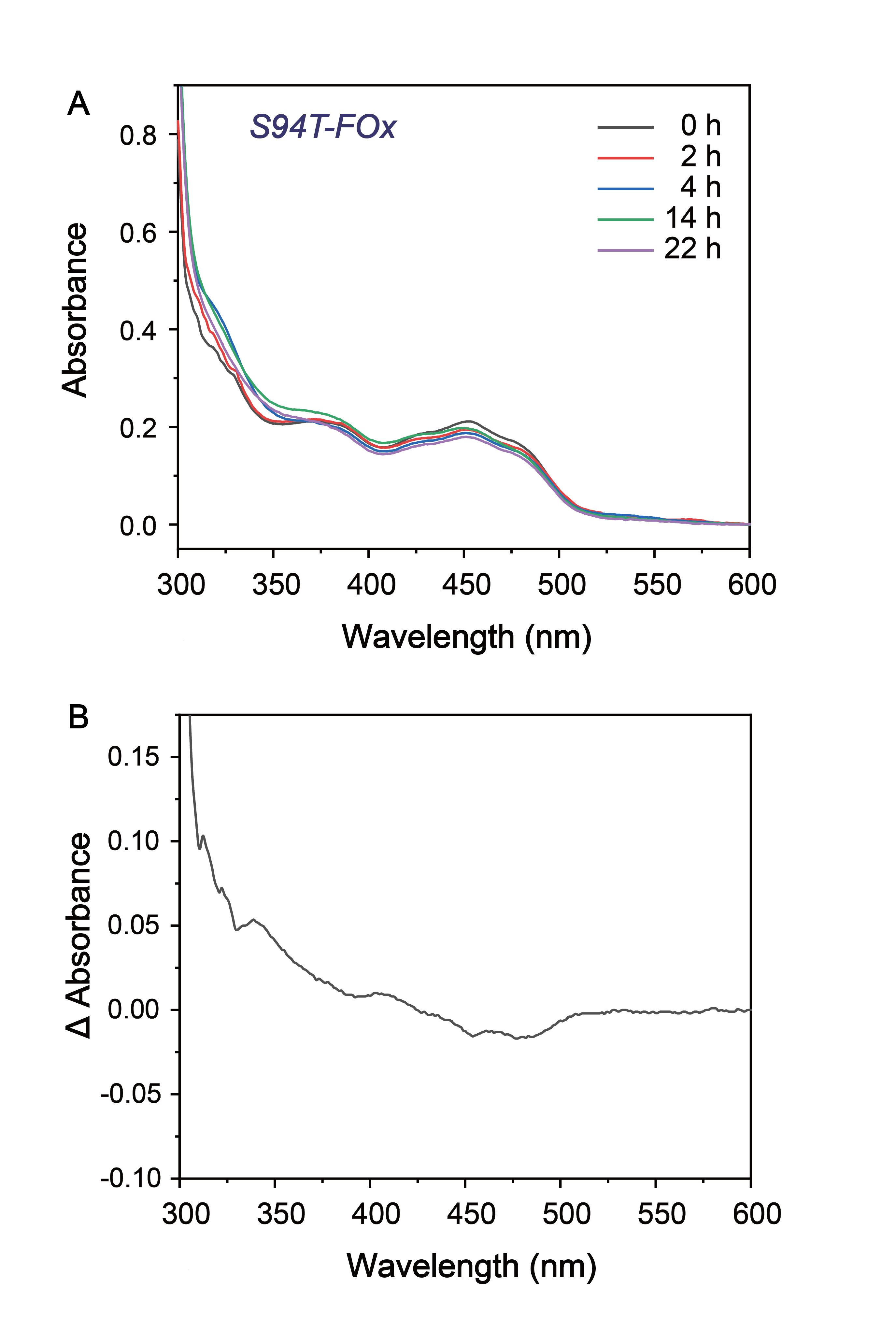
**

**Fig. S3.** Time dependence of 8-formyl FAD formation in S94T FOx. (**A**) UV-visible absorption spectra ranging from 300 to 600 nm were recorded at various time points: 0 h (black), 2 h (red), 4 h (blue), 14 h (green), and 22 h (purple) following cell lysis. (**B**) The changes in the absorption spectrum of S94T FOx between the initial measurement at 0 h and after 14 h post-cell lysis.

**Table S1.** Steady-state kinetic parameters of WT FOx at 0, 14, 24 and 36 h after the completion of protein purification.

| **WT FOx** | **0 h** | **14 h** | **24 h** | **36 h** |
| --- | --- | --- | --- | --- |
| *k*_cat_[s^-1^] | 78 ± 5.2 | 102 ± 5.7 | 90 ± 0.6 | 75 ± 2.3 |
| *K*_m_[mM] | 99 ± 12.4 | 96 ± 3.1 | 97 ± 12.1 | 80 ± 1.6 |
| *k*_cat_/*K*_m_[mM^−1^s^−1^] | 0.8 ± 0.05 | 1.1 ± 0.09 | 0.9 ± 0.12 | 0.9 ± 0.01 |

**Table S2.** Reaction coordinates in the mechanism of FOx-mediated oxidation of formate.

| **Reaction coordinates** | **Detailed setup** |
| --- | --- |
| RC1 | *d*(formate:H $\cdots$ 8-formyl FAD:N5) |
| RC1’ | *d*(formate:H $\cdots$ FAD:N5) |

**Table S3.** Reaction coordinates in the mechanism of self-catalytic conversion of FAD to 8-formyl FAD.

| **Reaction coordinates** | **Detailed setup** |
| --- | --- |
| RC1 | *d*(OH^-^:O $\cdots$ FAD:H_C8M_) |
| RC2 | *d*(O_2_:O $\cdots$ FAD_INT1_:C8) |
| RC3 | *d*(FAD_INT2_:O_C8M_ $\cdots$ H_2_O:H) |
| RC4 | *d*(H_2_O:O $\cdots$ FAD_INT2_:H_C8M_) |
| RC1’ | *d*(OH^-^:O $\cdots$ R87:HN) |
| RC2’ | *d*(R87:N $\cdots$ FAD:H_C8M_) |
| RC3’ | *d*(OH^-^:O $\cdots$ S94:HG) |
| RC4’ | *d*(S94:OG $\cdots$ FAD_INT1’_:C8) |
| RC5’ | *d*(FAD_INT1’_:N5 $\cdots$ H_2_O:H) |

**Table S4.** Steady-state kinetic parameters of R87K FOx at 0, 14, 24, 36 h after the completion of protein purification.

| **R87K FOx** | **0 h** | **14 h** | **24 h** | **36 h** |
| --- | --- | --- | --- | --- |
| *k*_cat_[s^-1^] | 51 ± 1.8 | 76 ± 0.1 | 76 ± 0.4 | 71 ± 2.1 |
| *K*_m_[mM] | 136 ± 13.0 | 135 ± 0.7 | 146 ± 5.0 | 148 ± 1.3 |
| *k*_cat_/*K*_m_[mM^−1^s^−1^] | 0.4 ± 0.02 | 0.6 ± 0.01 | 0.5 ± 0.02 | 0.5 ± 0.01 |

**Table S5.** Steady-state kinetic parameters of R87H FOx at 0, 14, 24, 36 h after the completion of protein purification.

| **R87H FOx** | **0 h** | **14 h** | **24 h** | **36 h** |
| --- | --- | --- | --- | --- |
| *k*_cat_[s^-1^] | 2 ± 0.2 | 3 ± 0.2 | 3 ± 0.2 | 11 ± 1.1 |
| *K*_m_[mM] | 84 ± 17.8 | 78 ± 6.6 | 81 ± 3.2 | 57 ± 11.9 |
| *k*_cat_/*K*_m_[mM^−1^s^−1^] | < 0.1 | < 0.1 | < 0.1 | 0.2 ± 0.02 |

**Table S6.** Steady-state kinetic parameters of S94I FOx at 0, 14, 24, 36 h after the completion of protein purification.

| **S94I FOx** | **0 h** | **14 h** | **24 h** | **36 h** |
| --- | --- | --- | --- | --- |
| *k*_cat_[s^-1^] | 2 ± 0.1 | 3 ± 0.1 | 13 ± 0.2 | 15 ± 1.5 |
| *K*_m_[mM] | 72 ± 7.2 | 77 ± 6.1 | 72 ± 5.5 | 61 ± 15.3 |
| *k*_cat_/*K*_m_[mM^−1^s^−1^] | < 0.1 | < 0.1 | 0.2 ± 0.01 | 0.3 ± 0.04 |

**Table S7.** Steady-state kinetic parameters of S94T FOx at 0, 14, 24, 36 h after the completion of protein purification.

| **S94T FOx** | **0 h** | **14 h** | **24 h** | **36 h** |
| --- | --- | --- | --- | --- |
| *k*_cat_[s^-1^] | 4 ± 0.1 | 4 ± 0.1 | 5 ± 0.2 | 5 ± 0.1 |
| *K*_m_[mM] | 82 ± 4.8 | 97 ± 9.0 | 90 ± 8.5 | 86 ± 2.5 |
| *k*_cat_/*K*_m_[mM^−1^s^−1^] | < 0.1 | < 0.1 | < 0.1 | < 0.1 |

**Table S8.** Primer sequences used for site-directed mutagenesis.

| **Primer** | **DNA sequence 5’-3’** |
| --- | --- |
| R87H-forward | GAATACCCATGGTAAAACCCTGGGTGGTAGC |
| R87H-reverse | ACCATGGGTATTCGGTTTTTCAATACGTTCA |
| R87K-forward | AAACCGAATACCAAAGGTAAAACCCTGGGTGGTAGCAGC |
| R87K-reverse | CCCAGGGTTTTACCTTTGGTATTCGGTTTTTTTCAATACGTT |
| S94I-forward | CTGGGTGGTATTAGCAGCCTGAACTATTTTACCTG |
| S94I-reverse | TCAGGCTGCTAATACCACCCAGGGTTTTACCA |
| S94T-forward | GTGGTACCAGCAGCCTGAACTATTTTACCTGGGTT |
| S94T-reverse | TTCAGGCTGCTGGTACCACCCAGGGTTTTAC |

**Table S9.** The structural models for MD simulation in this work.

| **Residue function study** | **FOx-mediated the oxidation of formate** | **Formation of 8-formyl FAD** |
| --- | --- | --- |
| - WT FOx/8-fFAD/OH^-^ - R87K FOx/8-fFAD/OH^-^ - R87H FOx/8-fFAD/OH^-^ - S94I FOx/8-fFAD/OH^-^ - S94T FOx/8-fFAD/OH^-^ | - WT FOx/FAD/formate - WT FOx/8-fFAD/formate | - WT FOx/FAD/OH^-^/O_2_/H_2_O |
