# Supplementary figures and images for: Mechanistic insights into the conversion of flavin adenine dinucleotide (FAD) to 8-formyl FAD in formate oxidase: a combined experimental and in-silico study
Source: Bioresour Bioprocess. 2024 Jul 10;11(1):67. doi: 10.1186/s40643-024-00782-4 (PMC11236828; doi:10.1186/s40643-024-00782-4)

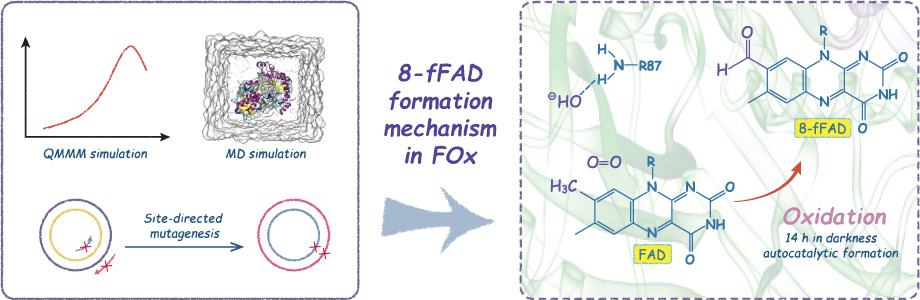

Supplement: Supplementary file 2 — Supplementary Material 2 [file 40643_2024_782_MOESM2_ESM.jpg]
